# The Functional Role and Prognostic Significance of TIM-3 Expression on NK Cells in the Diagnostic Bone Marrows in Acute Myeloid Leukemia

**DOI:** 10.3390/biomedicines12122717

**Published:** 2024-11-27

**Authors:** Kai Sun, Zong-Yan Shi, Dai-Hong Xie, Ya-Zhe Wang, Hao Jiang, Qian Jiang, Xiao-Jun Huang, Ya-Zhen Qin

**Affiliations:** Peking University People’s Hospital, Peking University Institute of Hematology, National Clinical Research Center for Hematologic Disease, Beijing Key Laboratory of Hematopoietic Stem Cell Transplantation, Beijing 100044, China; sunkai8322@163.com (K.S.); zy25816s@163.com (Z.-Y.S.); xiedaihong2022@163.com (D.-H.X.); ya_zhe2003@126.com (Y.-Z.W.); jiangha0090@sina.com (H.J.); jiangqian@medmail.com.cn (Q.J.); xjhrm@medmail.com.cn (X.-J.H.)

**Keywords:** acute myeloid leukemia, TIM-3, NK cells, prognostic significance, cell-killing

## Abstract

**Background:** Compared to other immune checkpoint molecules, T cell immunoglobulin domain and mucin domain-3 (TIM-3) is highly expressed on natural killer (NK) cells, but its functional role and prognostic significance in acute myeloid leukemia (AML) remains unclear. This study aims to evaluate the role of TIM-3 expression on the cytotoxic and killing capacity of NK cells and its prognostic significance in AML. **Methods:** AML public single-cell RNA sequencing (scRNAseq) data were used to analyze the correlation of transcript levels between *HAVCR2* (encoding TIM-3) and cytotoxic molecules in NK cells. NK cells from the bone marrows of seven newly diagnosed AML patients and five healthy donors (HDs) were stimulated in vitro and cell-killing activity was evaluated. A total of one hundred and five newly diagnosed adult AML patients and seven HDs were tested the expression of TIM-3 and cytotoxic molecules on the bone marrow NK cells by multi-parameter flow cytometry (MFC). **Results:** Both scRNAseq and MFC analysis demonstrated that TIM-3 expression on NK cells was positively related to the levels of perforin (PFP) and granzyme B (GZMB) (all *p* < 0.05) in AML. It was PFP and GZMB but not the TIM-3 level that was related to NK-cell-killing activity against K562 cells (*p* = 0.027, 0.042 and 0.55). A high frequency of TIM-3^+^ NK cells predicted poorer relapse-free survival (RFS) and event-free survival (EFS) (*p* = 0.013 and 0.0074), but was not an independent prognostic factor, whereas low GZMB levels in TIM-3^+^ NK cells independently predicted poorer RFS (*p* = 0.0032). **Conclusions:** TIM-3 expression on NK cells is positively related to PFP and GZMB levels but has no relation to cell-killing activity in AML, and low GZMB levels in TIM-3^+^ NK cells in the diagnostic bone marrows predicts poor outcomes. This study lays a theoretical foundation for the clinical application of immune checkpoint inhibitor treatment.

## 1. Introduction

Acute myeloid leukemia (AML) is a highly heterogeneous disease originating from the clonal proliferation of bone marrow (BM) immature myeloid cells [1]. It is the most common type of acute leukemia in adults with an incidence at 5.06 per 100,000 in western countries, and the average overall lifetime cost is up to CAD 478,516 [2,3]. The genetic and cytogenetic abnormalities constitute the framework for prognostic risk stratification of AML, and cytotoxic chemotherapy and allogeneic hematopoietic stem cell transplantation (allo-HSCT) shape the present main treatment landscape [4,5]. Although AML patients have benefited from the clinical application of allo-HSCT and novel targeted agents, the mortality rate is still high [1,6,7]. Therefore, it is necessary to draw novel therapies into the field of AML treatment.

In the past decade, cancer immunotherapy aimed at enhancing patients’ own active or passive anti-tumor immune response has changed the paradigm for cancer therapy [8]. T cells, which play a pivotal role in the adaptive immune response, are the primary target of cancer immunotherapy [9]. Compared to T cells, natural killer (NK) cells lack surface T-cell receptors (TCRs) and do not cause graft-versus-host disease (GVHD) due to the lack of MHC restriction [10,11]. In addition, the unique NK-cell receptors can sense tumor-related ligands and rapidly trigger the degranulation response and killing activity of NK cells [12,13]. Thus, NK cells are now becoming promising and ideal target for cancer immunotherapy. In addition to engineered T or NK cells and cancer vaccines, immune checkpoint inhibitor-based therapy is revolutionizing anti-tumor immunotherapy [8,14].

Compared to other immune checkpoint molecules, T cell immunoglobulin domain and mucin domain-3 (TIM-3) is highly expressed on NK cells with a median frequency of >50% in the human peripheral blood samples [13,15,16]. However, the mechanism by which TIM-3 affects the function of NK cells has not been clearly clarified. Ndhlovu et al. reported that increased frequency of TIM-3 on NK cells was related to stronger cytokine production and degranulation response but was associated with a suppressed direct killing activity of NK cells [17]. Folgiero et al. demonstrated that TIM-3^+^ NK cells interact with AML blasts expressing Galectin-9 (Gal-9), and inhibition of TIM-3 or Gal-9 affected NK cell-dependent IFN-γ production as well as the activation of IDO1 in AML blasts [18]. In contrast, Rakova et al. showed that AML patients with a high frequency of TIM-3 on NK cells had superior cytotoxicity against NK cell-sensitive K562 cells. Furthermore, they found that a high frequency of TIM-3^+^ NK cells correlated with improved survival [15]. To our knowledge, this was the only report evaluating the prognostic significance of TIM-3 expression on NK cells in AML. Therefore, clinical cohort studies with large sample sizes are needed to clarify the prognostic significance of TIM-3 expression on NK cells in AML.

In the present study, through single-cell RNA sequencing (scRNA-seq) analysis and fresh BM sample testing using multi-parameter flow cytometry (MFC), we investigated the correlation between NK cell TIM-3 expression and the levels of NK cell function related cytotoxic granules. Furthermore, through in vitro NK cell stimulation assays individually using BM mononuclear cells (BMMCs) and isolated NK cells, we evaluated the impact of TIM-3 expression and cytotoxic molecule synthesis on the direct killing activity of NK cells. Moreover, based on a large-scale cohort with follow-up, we thoroughly evaluated the prognostic significance of TIM-3 expression and the cytotoxic molecules of NK cells on patients’ outcomes.

## 2. Materials and Methods

### 2.1. scRNA-Seq Data Analysis of HAVCR2 (+) and HAVCR2 (−) NK Cells in AML

**Figure 1:**
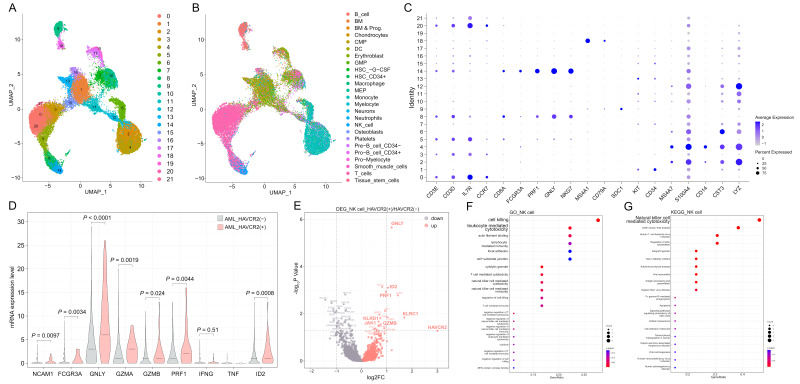
Dissection, clustering and function enrichment analysis based on *HAVCR2* (+) and *HAVCR2* (−) NK cells of AML patient BM cells. The UMAP projection of BM cells from sixteen AML patients, showing the formation of 22 main clusters (**A**). Cell type annotation by “SingleR” package, showing NK cells (marked with blue dots) in the clusters 14 and 8 (**B**). Dot plot of immune cells and leukemia stem cells related genes, identifying NK cells with *FCGR3A*, *PRF1*, *GNLY* and *NKG7* in clusters 14 and 8 (**C**). Gene Ontology (GO) and Kyoto Encyclopedia of Genes and Genomes (KEGG) enrichment analysis using the top 20 differentially expressed genes (DEGs) highly expressed in *HAVCR2* (+) versus *HAVCR2* (−) NK cells (**D**,**E**). Volcano plot of DEGs using the ratio of *HAVCR2* (+) and *HAVCR2* (−) of NK cells (**F**). Violin plot of the marker genes in NK cell maturation, degranulation, cytokine production and function regulation compared between *HAVCR2* (+) and *HAVCR2* (−) NK cells (**G**).

In van Galen et al.’s report, scRNA-seq data of sixteen AML patients were used. We downloaded them from Gene Expression Omnibus (GEO) database (GSE116256). All patients’ information is shown in Table S1 [19]. R package “Seurat” was used for data processing. The single-cell datasets were integrated using the “cbind” command and “harmony” function. Cells with unique feature counts >1500 or <200 as well as mitochondrial counts ≥ 5% were filtered. Data quality was showed in Figure S1A,B. The integrated dataset was normalized using Seurat functions “NormalizedData”, “FindVariableFeatures” and “ScaleData” with “LogNormalize” and “vst” methods. Seurat “JackStrawPlot” and “ElbowPlot” functions were used to determine appropriate dimensionality. The results of dimension-reduction analysis using seurat “RunUMAP” function were shown in Figure S1C and Figure 1A. R package “SingleR” was then used for cell type annotation, and “HumanPrimaryCellAtlasData” affiliated to R package “celldex” was used as the reference. The results of “SingleR” annotation and cluster identification based on “SingleR” were individually shown in Figure S4D and Figure 1B. The detailed patients’ information and analysis procedure referred to the reports individually by van Galen et al. and Guo et al. [19,20].

Considering the expression levels of NK cell markers “*FCGR3A*”, “*PRF1*”, “*GNLY*” and “*NKG7*” (individually encoding CD16a, Perforin, Granzyme G, and NKG7 proteins), NK cells were distributed in cluster 8 and cluster 14 (Figure 1C). Cells annotated as “NK cell” by “SingleR” in clusters 8 and 14 were isolated and finally identified as “NK cells” in the present study. Those with *HAVCR2* (encoding TIM-3 protein) expression levels equal to zero were designated as the *HAVCR2* (−) group, and those with *HAVCR2* expression higher than zero were designated as the *HAVCR2* (+) group.

R package “DESeq2” and function “DESeqDataSetFromMatrix” were used for differentially expressed gene (DEG) analysis between the *HAVCR2* (+) group and *HAVCR2* (−) group. |log2FC| ≥ 0.5 and *p* value < 0.05 were selected as DEGs. R packages “clusterProfiler”, “org.Hs.eg.db”, and “tidyverse” as well as functions “enrichGO” and “enrichKEGG” were used for functional enrichment analysis of the DEGs [21].

### 2.2. Clinical Cohort

A total of one hundred and five adult non-M3 AML patients who were consecutively diagnosed in our institute from September 2022 to May 2023 and had remaining BM samples after immunophenotyping testing at diagnosis were enrolled in the present study. The median age of all patients was 47 (range 16–65) years at diagnosis, and male patients accounted for 52.4% (55/105). The diagnosis was based on BM morphology, immunophenotyping, karyotyping, and molecular testing [22]. All patients were screened for common AML-related fusion transcripts, *NPM1* and *FLT3-ITD* mutations, as we previously described [23,24], and ninety of them were screened for AML-related gene mutations by targeted next-generation sequencing.

A total of seventy-seven (73.3%) enrolled patients received treatment as we described previously [25,26] and were followed up in our institute; forty-nine patients received IA (idarubicin and cytarabine) or HAA (homoharringtonine, aclarubicin and cytarabine), five patients received CAG (cytarabine, aclarubicin and G-CSF), sixteen received Azacitidine combined with Venetoclax, and five patients received IA, HAA or CAG combined with Azacitidine or targeted therapy as induction regimens, and the remaining two patients died before receiving induction chemotherapy. After achieving complete remission (CR), patients received medium or high dose cytarabine-based chemotherapy or chemotherapy followed by allo-HSCT as consolidation therapy. The detailed protocols for pre-conditioning regimens and graft-versus-host disease prophylaxis of allo-HSCT were comprehensively reported in our previous reports [27]. The cut-off date for the last follow-up was April 2024.

This research was approved by the Ethics Committee of the Peking University People’s Hospital and was in accordance with the Declaration of Helsinki.

### 2.3. Flow Cytometric Analysis of TIM-3 and Cytotoxic Molecules Expression on NK Cells in Fresh BM Samples

Fresh BM specimens collected from one hundred and five newly diagnosed adult non-M3 AML patients and seven healthy donors (HDs) were tested TIM-3 as well asperforin (PFP) and granzyme B (GZMB) levels by MFC. Experimental procedures were as follows. First, 2 mL PBS was used to wash 1 × 10^6^ cells. Then, a 10 uL staining buffer was added to stabilize the cytomembranes, followed by fluorescein coupled antibody incubation with CD45-V500 (Biolegend, San Jose, CA, USA, Clone HI30), CD3-APC-H7 (BD Biosciences, Clone SK7), CD56-FITC (BD Biosciences, Clone NCAM1) and TIM-3-APC (Biolegend, Clone F38.2E2) for 15 min in dark. Subsequently, 100 uL buffer A of a Fix&Perm Cell Permeabilization kit (BD Biosciences, San Jose, CA, USA) was used to fix the cytomembranes for 5 min, and then FACS lysis solution (BD Biosciences, San Jose, CA, USA) was applied to lysing red blood cells for 8 min in dark. An amount of 50 uL buffer B of the Fix&Perm Cell Permeabilization kit (BD Biosciences, San Jose, CA, USA) was used to permeabilize the cytomembranes, followed by intracellular antibody incubation PFP-PerCP-Cy5.5 (Biolegend, Clone B-D48) and GZMB-BV421 (Biolegend, Clone QA18A28) for 20 min in dark. Finally, cells were washed by PBS, filtered by cell strainers, resuspended with 500 uL PBS and kept at 4 °C until acquisition. Navios (Beckman Coulter Life Sciences, Indianapolis, IN, USA) was used for cell acquisition, and Kaluza 2.0 (Beckman Coulter, Brea, CA, USA) was used for data analysis.

The gating strategy was as follows. After excluding doublets and removing cell debris, cells were defined as nucleated cells (NCs). Lymphocytes were defined as CD45^high^/SS INT^low^ in NCs, and NK cells were defined as CD3^−^CD56^+^ in lymphocytes. Positive TIM-3, PFP and GZMB cells in NK cells were circled out referring to their individual internal controls (Figure S2A,B). All gates for the same molecule were interrelated (Figure S2C). The proportions of TIM-3^+^, PFP^+^, GZMB^+^ and PFP^+^GZMB^+^ cells in NK cells were designated as their individual expression levels. The levels of PFP in TIM-3^+^ and TIM-3^−^ NK cells were calculated as PFP^+^TIM-3^+^/(PFP^+^TIM-3^+^+PFP^−^TIM-3^+^) and PFP^+^TIM-3^−^/(PFP^+^TIM-3^−^+PFP^−^TIM-3^−^), which was the same for GZMB.

### 2.4. In Vitro NK Cell Stimulation and Cell-Killing Activity Testing

BMMCs of seven newly diagnosed AML patients and five HDs were isolated from fresh BM specimens using Ficoll–Hypaque solution (TBDsciences, Tianjin, China), with centrifugation at a rotated speed of 2000 rpm and a deceleration of 0. Then, BMMCs were counted to 1 × 10^6^/mL and cultured with RPMI 1640 medium (Gibco, Grand Island, NY, USA) containing 10% fetal bovine serum (Gibco, Grand Island, NY, USA), and NK cell-sensitive K562 cells were counted to 2 × 10^5^/mL with a ratio of BMMCs: K562 cells at 5:1 to stimulate NK cells for 6 h. An amount of 1000 IU/mL IL-2 (CR PHARMA, Beijing, China) was supplemented to maintain the activity of lymphocytes. Protein Transport Inhibitor Cocktail (Invitrogen, Carlsbad, CA, USA) was added to inhibit NK cell degranulation for the testing of NK cell cytotoxic molecule.

After co-culture, cells for NK cell cytotoxic molecule testing were incubated with CD45-V500 (Biolegend, Clone HI30), CD3-APC-H7 (BD Biosciences, Clone SK7), CD56-FITC (BD Biosciences, Clone NCAM1) and TIM-3-APC (Biolegend, Clone F38.2E2), as well as PFP (Biolegend, Clone B-D48), GZMB-PE (Biolegend, Clone QA18A28) and IFN-γ (Biolegend, Clone B27) after cell fixation and permeabilization. Cells for NK-cell-killing activity testing were incubated with CD45-V500 (Biolegend, Clone HI30) as well as a cell apoptosis detection kit (RIBOBIO, Guangzhou, Chian) with Annexin-V (Alexa Fluor 647) and 7-AAD. Detailed flow cytometry procedures were similar as described above.

The gating strategy and calculation method of TIM-3, PFP, GZMB and IFN-γ for NK cell function testing were consistent with that in the fresh BM testing in Figure S2. In addition, the gating strategy for NK-cell-killing activity was shown in Figure S3. K562 cells were identified from BMMCs by their distinct cell size and granularity (Figure S3A). Then, FLT4 and FLT6 of Navios was individually applied to detect 7-AAD and Annexin-V-positive cells. The apoptosis cells were calculated as the sum of cell proportions added in Q2 and Q3 (Figure S3B).

### 2.5. Sorting of TIM-3^+^ and TIM-3^−^ NK Cells and In Vitro NK Cell Function Testing

Fresh BMMCs were used for fluorescence-activated cell sorting. First, cells were counted to a total of >5 × 10^7^ in order to collect enough cells. Then, fluorescein coupled antibodies CD45-V500 (Biolegend, Clone HI30), CD3-APC-H7 (BD Biosciences, Clone SK7), CD56-FITC (BD Biosciences, Clone NCAM1) and TIM-3-APC (Biolegend, Clone F38.2E2) were used to label TIM-3^+^ and TIM-3^−^ NK cells. BD FACS Aria SORP (BD Biosciences, San Jose, CA, USA) was used for cell sorting. The gating strategy for fluorescence-activated cell sorting and cell examination are shown in Figure S4. The purity of both TIM-3^−^ and TIM-3^+^ NK cells were approximately 80%.

After cell sorting, a co-culture system was established with a ratio of NK cells: K562 cells at 2.5:1. Then, IL-2 (CR PHARMA, Beijing, China) was added for maintaining the activity of lymphocytes, with or without an application of a Protein Transport Inhibitor Cocktail (Invitrogen, Carlsbad, CA, USA) individually for NK cell cytotoxic molecule testing and cell-killing activity testing. PFP, GZMB and IFN-γ were tested for NK cell cytotoxic molecule production, and K562 cell apoptosis was evaluated in the NK-cell-killing activity assay. The gating strategy was similar to what has been mentioned above.

### 2.6. Definitions and Statistical Analysis

Complete remission (CR) was defined as morphologic CR. Relapse-free survival (RFS) was measured from CR to relapse, or to the last date of BM morphological assessment. The events of event-free survival (EFS) included not achieving CR after 2-course induction, relapse and death from any cause. EFS was measured from diagnosis to not achieving CR after 2-course induction or death, or from CR to relapse or to the last date of BM morphological assessment.

The Mann–Whitney *U* test and *Fisher*’s exact test were individually used for pairwise comparisons of continuous variables and categorical variables. The paired *t* test or Wilcoxon rank sum test were used for comparisons between paired samples. The Kaplan–Meier method and log-rank tests were used for the survival function estimate. Variables associated with *p* < 0.20 in the univariate analysis were entered into Cox model-based multivariable analysis. *p* values less than 0.05 were considered statistically significant. SPSS 26.0 software package (SPSS Inc., Chicago, IL, USA) and GraphPad Prism 9 (GraphPad Software Inc., La Jolla, CA, USA) were used for data analysis.

## 3. Results

### 3.1. scRNA-Seq Data Indicated the Positive Correlation of Transcript Levels Between HAVCR2 and Cytotoxic Molecules in the BM NK Cells

Through comparing the AML scRNA-seq data of *HAVCR2* (+) with *HAVCR2* (−) NK cells, signaling pathways involving in cell-killing and leukocyte mediated cytotoxicity of Gene Ontology (GO) analysis as well as natural killer cell mediated cytotoxicity of Kyoto Encyclopedia of Genes and Genomes (KEGG) analysis were the most significantly enriched using the top 20 DEGs of up-regulated genes (Figure 1D,E). DEGs were shown using the volcano plot, and the transcript levels of NK cell cytotoxicity and killing receptors related *GNLY*, *ID2*, *PRF1*, *KLRC1*, *KLRD1*, *GZMB* and *JAK1* were significantly higher in *HAVCR2* (+) NK cells (Figure 1F). *HAVCR2* expression was related to higher transcript levels of NK cell markers *NCAM1* (encoding CD56 protein) and *FCGR3A* (encoding CD16a protein) (*p* = 0.0097 and 0.0034), cytotoxic granule genes *GNLY*, *GZMA*, *GZMB* and *PRF1* (encoding perforin protein) (*p* < 0.0001, = 0.0019, 0.024 and 0.0044), as well as immune cell differentiation related gene *ID2* (*p* = 0.0008), whereas the transcript levels of cytokine related genes, *IFNG* (encoding IFN-γ protein) and *TNF* (encoding TNF family proteins) were low and did not show significant differences between *HAVCR2* (+) and *HAVCR2* (−) NK cells, respectively (Figure 1G). These results indicated that *HAVCR2* expression positively correlated with the transcript levels of cytotoxic molecules in NK cells.

### 3.2. TIM-3 Expression Correlated with PFP and GZMB Expression in Fresh BM NK Cells

**Figure 2:**
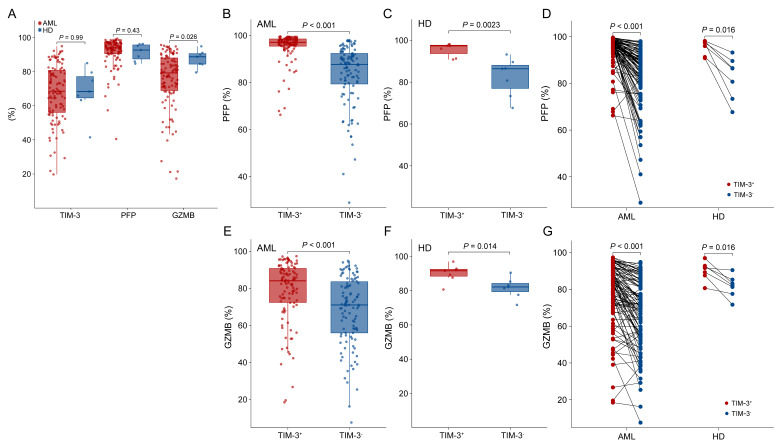
TIM-3, Perforin (PFP) and Granzyme B (GZMB) levels of NK cells and their interrelationships in AML and healthy donors (HDs) using fresh bone marrow (BM) samples. The general comparisons of TIM-3, PFP and GZMB levels between AML patients and HDs (**A**). General grouped comparison charts of the PFP and GZMB levels in NK cells by distinguishing TIM-3^+^ and TIM-3^−^ individually in AML (**B**,**E**) and HDs (**C**,**F**). Paired comparison plots of the PFP (**D**) and GZMB (**G**) levels in NK cells of a single person by distinguishing TIM-3^+^ and TIM-3^−^.

The expression patterns of NK cell TIM-3, PFP and GZMB in fresh BM samples of AML patients and HDs tested by MFC are shown in Figure 2A. The TIM-3 and PFP levels of NK cells were similar between AML patients and HDs (median (range): 68.9% (19.7–95.0%) vs. 68.4% (41.5–85.0%), *p* = 0.99; 94.1% (40.5–99.4%) vs. 92.7% (84.7–96.3%), *p* = 0.43), whereas AML patients had significantly lower GZMB levels than HDs (median (range): 79.5% (17.3–95.7%) vs. 88.8% (79.6–94.9%), *p* = 0.026).

Both AML patients and HDs showed significantly higher PFP levels in TIM-3^+^ NK cells than TIM-3^−^ cells (*p* < 0.001 and =0.0023, Figure 2B,C). A pairwise analysis of TIM-3^+^ to TIM-3^−^ NK cells of individuals of both AML patients and HDs showed consistent results (*p* < 0.001 and =0.016, Figure 2D). Similarly, TIM-3^+^ NK cells showed significantly higher GZMB levels than TIM-3^−^ NK cells in both AML patients and HDs (*p* < 0.001 and =0.014, Figure 2E,F), and pairwise analysis showed consistent results (*p* < 0.001 and =0.016, Figure 2G).

### 3.3. The Relationship Between TIM-3 Expression and PFP, GZMB and IFN-γ Levels in NK Cells After In Vitro Stimulation

**Figure 3:**
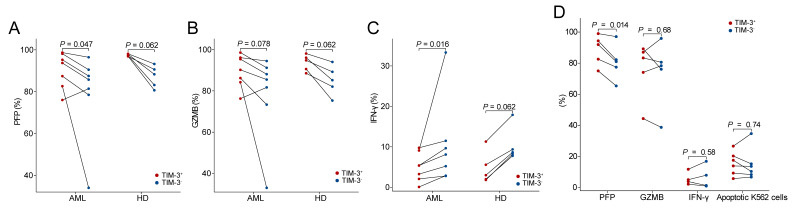
The relationship between TIM-3 expression and PFP, GZMB and IFN-γ levels in NK cells after in vitro stimulation. Paired comparison of TIM-3^+^ to TIM-3^−^ cells of the PFP (**A**), GZMB (**B**) and IFN-γ (**C**) levels between TIM-3^+^ and TIM-3^−^ NK cells in BMMCs. Paired comparison of the PFP, GZMB, IFN-γ and apoptotic K562 cell proportion between TIM-3^+^ and TIM-3^−^ NK cells isolated from AML BMMCs (**D**).

Consistent with the results from the scRNA-seq data and fresh BM samples with no stimulation, TIM-3^+^ NK cells had higher intracellular PFP and GZMB levels than TIM-3^−^ NK cells in AML patients’ BMMCs after in vitro stimulation (*p* = 0.047 and 0.078), and HDs showed similar trends (both *p* = 0.062, Figure 3A,B). In contrast, the cytoplasmic IFN-γ level was significantly higher in TIM-3^−^ than that in TIM-3^+^ NK cells in AML patients, and the same tendency existed in HDs (*p* = 0.016 and 0.062, Figure 3C).

TIM-3^+^ and TIM-3^−^ NK cells were isolated from AML BMMCs and separately co-cultured with K562 cells. TIM-3^+^ cells showed significantly higher intracellular PFP levels than TIM-3^−^ NK cells (*p* = 0.014), whereas they showed similar GZMB and IFN-γ levels to TIM-3^−^ NK cells (*p* = 0.68 and 0.58, Figure 3D).

### 3.4. In Vitro NK-Cell-Killing Capacity of AML Patients Was Not Related to TIM-3 Expression

**Figure 4:**
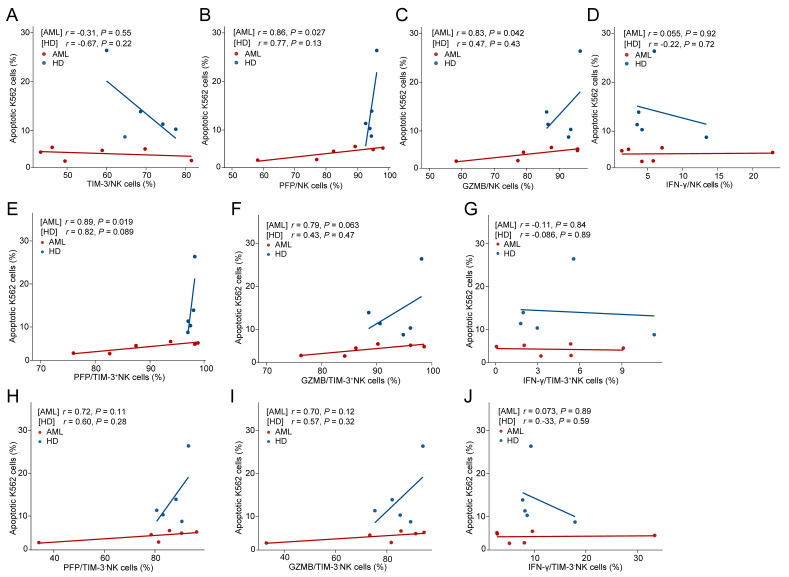
Testing of in vitro NK-cell-killing capacity versus K562 cells after stimulation using BMMCs. The correlations between in vitro NK-cell-killing capacity versus K562 cells and the levels of TIM-3 (**A**), PFP (**B**), GZMB (**C**) as well as IFN-γ (**D**) of NK cells in AML patients (red line) and HDs (blue line) after stimulation using BMMCs. Correlation analysis between K562 apoptosis proportion and PFP (**E**,**H**), GZMB (**F**,**I**) and IFN-γ (**G**,**J**) levels individually in TIM-3^+^ and TIM-3^−^ NK cells in AML patients (red line) and HDs (blue line).

We evaluated NK-cell-killing capacity against K562 cells after in vitro stimulation of BMMCs (Figure 4). The TIM-3 levels on NK cells did not show a significant correlation with the proportion of apoptotic K562 cells in both AML patients and HDs (*r* = −0.31, *p* = 0.55; *r* = −0.67, *p* = 0.22, Figure 4A). Furthermore, when TIM-3^+^ and TIM-3^−^ NK cells were separated from AML BMMCs, there was still no significant difference in the proportion of apoptotic K562 cells between them (*p* = 0.74, Figure 3D).

### 3.5. In Vitro NK-Cell-Killing Capacity of AML Patients Positively Correlated with PFP and GZMB Levels

For BMMCs after in vitro stimulation, the proportion of apoptotic K562 cells was significantly positively correlated with PFP and GZMB levels of NK cells in AML (*r* = 0.86, *p* = 0.027, and *r* = 0.83, *p* = 0.042, respectively, Figure 4B,C), and showed a positive correlation trend with PFP levels of NK cells in HDs (*r* = 0.77, *p* = 0.13, Figure 4B), but did not show a significant difference with GZMB levels in HDs (*p* = 0.43, Figure 4C). In addition, neither AML patients nor HDs showed significant correlations between the proportion of apoptotic K562 cells and IFN-γ levels (*p* = 0.92 and 0.72, Figure 4D).

In accordance with total NK cells, PFP and GZMB levels exhibited a positively correlated tendency with K562 cell apoptosis in both TIM-3^+^ and TIM-3^−^ NK cells in AML patients, and these correlations were more significant in TIM-3^+^ than in TIM-3^−^ NK cells (TIM-3^+^: *r* = 0.89, *p* = 0.019; *r* = 0.79, *p* = 0.063; TIM-3^−^: *r* = 0.72, *p* = 0.11; *r* = 0.70, *p* = 0.12, Figure 4E,F,H,I). However, in HDs, only PFP in TIM-3^+^ NK cells showed a positive correlation tendency with the apoptotic K562-cell proportions, but no correlation existed for GZMB levels in TIM-3^+^ NK cells and for PFP and GZMB levels in TIM-3^−^ NK cells (TIM-3^+^: *r* = 0.82, *p* = 0.089; *r* = 0.43, *p* = 0.47; TIM-3^−^: *r* = 0.60, *p* = 0.28; *r* = 0.57, *p* = 0.32, Figure 4E,F,H,I). IFN-γ levels in neither TIM-3^+^ nor TIM-3^−^ cells showed a correlation with K562 apoptosis in either AML or HDs (all *p* > 0.05, Figure 4G,J).

### 3.6. Outcomes and Clinical Characteristics of Follow-Up Patients

Of all one hundred and five patients enrolled, seventy-seven (73.3%) patients were followed up for a median of 12.6 (range: 0.2–19.7) months. In total, seventy (90.9%) patients achieved CR after induction treatment. Among them, fifty-nine (76.6%), ten (10/77, 13.0%) and one (1/77, 1.3%) patient achieved CR individually after 1-, 2- and >3-course induction chemotherapy. After CR achievement, fifty-one patients received chemotherapy as consolidation therapy, seventeen (33.3%) of which experienced subsequent relapse and six of them received allo-HSCT as rescue treatment. The remaining nineteen patients received chemotherapy followed by allo-HSCT as consolidation therapy (matched sibling donor, *n* = 3; haploidentical related donor, *n* = 15; matched unrelated donor, *n* = 1), and two (10.5%) patients relapsed. The 2-year RFS and EFS rates were 64.8% (95% confidence interval (CI), 47.2–77.9%) and 55.0% (95% CI, 40.0–68.3%), respectively.

Patients’ baseline demographic and clinical characteristics and their relationship with BM NK cell TIM-3 expression were summarized in Table 1. Higher platelet counts and adverse ELN genetic risk classification tended to associate with high TIM-3 expression on NK cells (*p* = 0.091 and 0.094, respectively), but other demographic and clinical characteristics did not significantly relate to TIM-3 expression (all *p* > 0.05).

### 3.7. High TIM-3 and Low GZMB Levels of NK Cells at Diagnosis Predicted Poorer RFS in AML

The prognostic significance of TIM-3, PFP and GZMB levels of BM NK cells in newly diagnosed AML patients was evaluated. Firstly, we used the quartile of each indicator to preliminarily suggest the relationship between each indicator and patients’ survival. The TIM-3 and GZMB levels of NK cells showed a related tendency with RFS (*p* = 0.15 and 0.10, respectively, Figure S5A,C), but that of PFP levels did not relate to RFS (*p* = 0.75, Figure S5B). In addition, the level of TIM-3 on NK cells tended to be associated with EFS (*p* = 0.12, Figure S5D), but both PFP and GZMB levels of NK cells were not related to EFS (both *p* = 0.42, Figure S5E,F). Thereafter, we performed ROC analysis to determine the optimal cutoff values for these indicators related to patients’ survival. Relapse or event occurrence was individually served as the outcome variable for RFS or EFS, and ROC analysis with AUC > 0.60 and *p* value < 0.20 was considered significant. Then, the optimal cutoff value was calculated according to the maximum Youden index. Indicators TIM-3 and GZMB levels of NK cells determined by relapse as well as the TIM-3 levels on NK cells determined by event occurrence all obtained appropriate values, which were 72.0%, 77.2% and 75.0%, respectively (Figure S6A–C).

**Figure 5:**
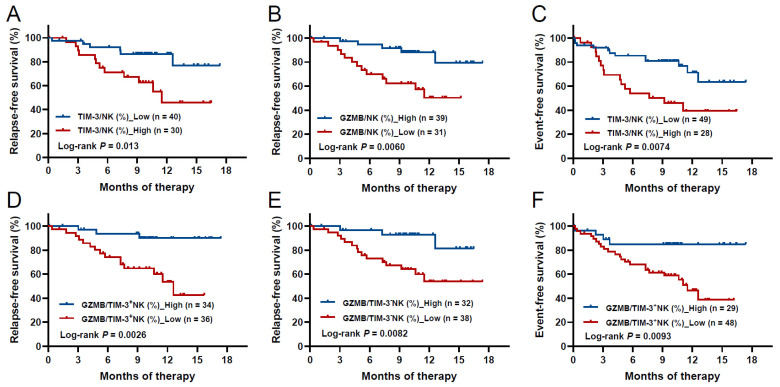
The Kaplan–Meier survival curves of AML patients based on variables with optimal cut-off values. The RFS curves of TIM-3 expression on NK cells (**A**) and GZMB levels in NK cells (**B**), and the EFS curve of TIM-3 expression on NK cells (**C**). The RFS curves of GZMB levels in TIM-3^+^ (**D**) and TIM-3^−^ (**E**) NK cells, and the EFS curve of GZMB levels in TIM-3^+^ NK cells (**F**).

Univariate analysis showed that high TIM-3 and low GZMB levels of NK cells at diagnosis were significantly related to poorer RFS (2-year RFS rate: 45.8% (95% CI: 21.4–67.4%) vs. 77.0% (95% CI: 48.9–90.9%), *p* = 0.013; 50.4% (95% CI: 28.4–68.8%) vs. 79.4% (51.9–92.2%), *p* = 0.0060, respectively, Table 2, Figure 5A,B). In addition, ELN genetic intermediate risk and chemotherapy alone as consolidation therapy were significantly related to poorer RFS (2-year RFS rate: 55.1% (95% CI: 25.5–77.1%) vs. 71.7% (95% CI: 41.7–88.1%, *p* = 0.030; 56.8% (95% CI: 35.0–73.8%) vs. 84.0% (95% CI: 46.8–96.0%), *p* = 0.038, respectively). Multivariate analysis showed that low GZMB levels of NK cells, ELN genetic intermediate and adverse risks and chemotherapy only as consolidation therapy were independent poor RFS prognostic indicators (HR: 8.1 (95% CI: 2.2–30.7), *p* = 0.0020; 11.3 (95%CI: 2.9–44.9), *p* = 0.0006; 3.7 (95% CI: 1.1–12.9), *p* = 0.040; 12.9 (95% CI: 2.5–65.8), *p* = 0.0020, respectively, Table 2), whereas higher TIM-3 levels on NK cells could not independently predict poor RFS (*p* = 0.55).

In addition, univariate analysis showed that high TIM-3 levels of NK cells at diagnosis was significantly related to poorer EFS (2-year EFS rate: 39.3% (95% CI: 19.7–58.5%) vs. 63.4% (95% CI: 39.6–79.8%), *p* = 0.0074, Table 3, Figure 5C). High platelet counts, ELN-intermediate and adverse genetic risks, and receiving other non-intensive chemotherapy regimens as induction therapy were significantly related to poorer EFS (2-year EFS rate: 47.0% (95% CI: 27.8–64.1%) vs. 62.3% (95% CI: 33.8–81.4%), *p* = 0.035; 43.0% (95% CI: 18.9–65.3%) vs. 71.7% (95% CI: 41.7–88.1%), *p* = 0.0011; 38.8% (95% CI: 15.1–62.2%) vs. 71.7% (95% CI: 41.7–88.1%), *p* = 0.0061; 20.0% (95% CI: 0.8–58.2%) vs. 50.4% (95% CI: 29.5–68.0%), *p* = 0.0006, respectively). Multivariate analysis showed that ELN-intermediate and adverse genetic risks and receiving other non-intensive chemotherapy regimens were independent poor prognostic prediction indicators for EFS (HR: 4.2 (95% CI: 1.4–12.5), *p* = 0.0087; 6.4 (95%CI: 2.2–18.4), *p* = 0.0005; 5.6 (95% CI: 1.7–18.2), *p* = 0.0046), whereas receiving induction therapy with Azacitidine + Venetoclax was an independent protective factor for EFS compared to receiving intensive chemotherapy (HR: 0.1 (95% CI: 0.01–0.8), *p* = 0.027, Table 3). The levels of TIM-3 on NK cells could not independently predict EFS.

### 3.8. Low GZMB Levels in TIM-3^+^ NK Cells Predicted Poorer RFS Superior to GZMB Levels in Total NK Cells

We further evaluated the prognostic significance of PFP and GZMB levels in TIM-3^+^ and TIM-3^−^ NK cells, respectively. Similarly, the quartile of each indicator was used to preliminarily suggest the tendency between each indicator and RFS or EFS (Figure S5G–N). PFP levels in TIM-3^−^ NK cells, GZMB levels in TIM-3^+^ and TIM-3^−^ NK cells were significantly related to or tended to be associated with RFS (*p* = 0.022, 0.011 and 0.12, respectively, Figure S5H–J), and PFP levels in TIM-3^−^ NK cells as well as GZMB levels in TIM-3^+^ NK cells were related to EFS (*p* = 0.041 and 0.056, respectively, Figure S5L,M). ROC analysis for the above indicators was performed. Indicators of GZMB levels in TIM-3^+^ and TIM-3^−^ NK cells determined by relapse as well as GZMB levels in TIM-3^+^ NK cells determined by event occurrence obtained appropriate cutoff values (all AUC > 0.60 and *p* < 0.20, Figure S6E,F,H), which were 84.0%, 72.0% and 87.95%, respectively. Indicators of PFP levels in TIM-3^−^ NK cells determined by both relapse and event occurrence did not obtain appropriate cutoff values (Figure S6D,G).

Low GZMB levels in both TIM-3^+^ and TIM-3^−^ NK cells were significantly related to lower RFS rates (2-year RFS rates: 42.8% (95% CI: 18.9–64.9%) vs. 89.9% (95% CI: 71.9–96.7%), *p* = 0.0026; 53.9% (95% CI: 33.8–70.3%) vs. 81.3% (95% CI: 44.7–94.8%), *p* = 0.0082, respectively, Table 2, Figure 5D,E). The low GZMB level in TIM-3^+^ NK cells was significantly related to a lower EFS rate (2-year EFS rate: 38.8% (95% CI: 19.7–57.5%) vs. 85.0% (64.7–94.1%), *p* = 0.0093, Table 3, Figure 5F). Multivariate analysis showed that low GZMB levels in TIM-3^+^ NK cells replaced GZMB levels in total NK cells as a superiorly independent prognostic factor for RFS (HR: 7.7 (95% CI: 2.0–30.0), *p* = 0.0032). In addition, ELN risk category by genetics and consolidation therapy were also independent predictors for RFS (Table 2). However, neither TIM-3 expression on NK cells nor GZMB levels in TIM-3^+^ NK cells could independently predict EFS (*p* = 0.31 and 0.072, respectively, Table 3).

## 4. Discussion

Unlike classical checkpoint molecules such as PD-1 which is associated with suppressed T-cell anti-tumor immunity and caused poor clinical outcomes in AML [28,29,30,31], TIM-3 molecule expression on various cell types showed multi-functional performance due to the complex intracellular signaling. Although the TIM-3 molecule was initially identified on IFN-γ-producing CD4^+^ and CD8^+^ T cells [32], other cell types such as myeloid cells, innate immune cells like NK cells and mast cells have shown its expression [17,33,34]. TIM-3 is highly expressed in various solid tumors such as glioma, non-small cell lung cancers and hepatocellular carcinoma, and is associated with poor prognosis [35,36,37]. In particular, TIM-3 expressed on leukemia cells and was considered to be a marker of leukemia stem cells (LSCs) [38,39]. Thus, the application of TIM-3 inhibitor might simultaneously act on immune cells and LSCs, which has attracted more attention in leukemia-related research. By using starting cells from umbilical cord blood NK-derived induced pluripotent stem cells (iPSCs), Klaihmon et al. successfully established a single-cell clone of CAR-TIM3 iPSCs with enhanced anti-tumor activity against TIM3-positive AML cells compared with wild-type NK-like cells from parental iPSCs [40]. However, recently published clinical trial results of Sabatolimab (anti-TIM-3 monoclonal antibody) in combination with a hypomethylating agent (HMA) did not demonstrate a more promising efficacy with a CR rate of 22% versus the HMA only control group of 18% in previously untreated patients with high-risk myelodysplastic syndromes (HR-MDS) [41]. In another Phase Ib clinical trial, the overall CR rate of Sabatolimab+HMA treatment in HR- and very high-risk (vHR)-MDS patients was only 19.6% (10/53) [42]. These data threw out a demand for further exploration of the role of TIM-3 expression in the BM microenvironment of AML.

In the present study, through scRNA-seq analysis, fresh BM sample testing and in vitro stimulation, we demonstrated that TIM-3 levels on NK cells positively correlated with the levels of cytotoxic molecules PFP and GZMB. Although these results seem to contradict the view that TIM-3 functions as a co-inhibitory immune checkpoint molecule, they are consistent with several reports. Ndhlovu et al. demonstrated that TIM-3^+^ NK cells were related to higher CD107a levels compared with TIM-3^−^ NK cells in peripheral blood mononuclear cells (PBMCs) of HDs [17]. In Rakova et al.’s report, both RNA sequencing and MFC testing using PBMCs illustrated that AML patients with TIM-3^high^ on NK cells displayed significantly higher GZMB levels than those with TIM-3^low^ [15]. However, in the report by Yu et al., TIM-3 mediated suppression of CD107a degranulation in NK cells during the infection immunity [43]. Discussions about whether TIM-3 functions as a co-inhibitory or co-stimulatory molecule have been ongoing. These contradictory results might be attributed to the lack of known inhibitory signaling motifs of the TIM-3 molecule in its cytoplasmic tail, and TIM-3 loss-of-function mutations in human diseases would help further clarify the role of the TIM-3 molecule [44].

Using in vitro stimulated BMMCs, we found that the synthesis of cytotoxic molecules positively correlated with the direct killing capacity of NK cells. Furthermore, both BMMCs and isolated NK cells showed that TIM-3 expression was not related to NK cell direct killing capacity. Differing from us, Rakova et al. once isolated total NK cells from AML BMMCs and demonstrated that patients with TIM-3 ^high^ NK cells showed stronger K562 cell-killing activity than those with TIM-3 ^low^ [15]. Ndhlovu et al. reported that TIM-3^+^ NK cells were related to high cytokine production and degranulation, and TIM-3 cross-linking inhibited the killing activity versus K562 cells of NK cells in HDs [17]. They interpreted that TIM-3 expression was marked as mature and the most responsive subset of NK cells, but once encountered corresponding ligands, it would restrain the killing potential like other NK cell inhibitory receptors such as the inhibitory KIRs. Several NK-cell receptors have been found expressed on aging T cells, labeled as senescent effector T cells [45,46]. These results indicated that TIM-3 marks fully functional and responsive NK cells but also signals senescence after performance completion. In addition, the research on T-cell showed that co-expression of PD-1, LAG-3 and TIM-3 was associated with prominent activation (CD69 expression) and effector function (GZMB level), as well as elevated levels of proapoptotic markers in non-small cell lung cancer [47]. They interpreted that these results were inconsistent with a model that co-inhibitory receptors were up-regulated upon T-cell stimulation, in order to limit excessive responses and potential tissue damage. With regard to NK cells, Wu et al. illustrated that up-regulation of TIM-3 was related to both higher apoptosis and activation frequency of NKT cells in infection diseases [48]. Similarly, high TIM-3 expression on NK cells was related to increased NK cell apoptosis in Type 2 diabetes [49]. Tumor infiltrating TIM-3^+^ NK cells were also found to be more susceptible to apoptosis than TIM-3^−^ NK cells in the esophageal cancer [50]. Thus, we suppose that NK cells develop the capacity for cytokine production and degranulation during maturation and simultaneously obtaining the phenotype of TIM-3 expression; however, once TIM-3 is expressed, its binding to ligands will lead the activated NK cells to dysfunction and apoptosis.

Using isolated TIM-3^+^ and TIM-3^−^ NK cells from BMMCs, we could not demonstrate correlations between TIM-3 expression and the levels of GZMB and IFN-γ. We consider that these inconsistencies with BMMCs might be due to the existence of complex interactions between NK cells and leukemia cells in the AML BM microenvironment, and that NK cell maturation and functional performance depend on the binding of TIM-3 with its ligands.

Based on a follow-up clinical cohort, we found that high TIM-3 expression on NK cells was related to poor clinical outcomes in AML, but not an independent prognostic factor. It is consistent with the view that TIM-3 functions as a co-inhibitory immune checkpoint molecule in AML on NK cells, similar to the role of PD-1 and TIGIT in previous studies [51,52,53], and coincides with the reports of the role of TIM-3 expression on NK cells in other tumor types [54,55]. Notably, because TIM-3 expression correlates with higher cytotoxic molecule levels, this result seems to be contradictory with enhanced killing activity and a favorable prognosis of higher PFP and GZMB levels. We suppose this could be interpreted as “immune checkpoint molecules express and bind to the ligands to limit exaggerated responses and potential tissue damage” upon NK cell stimulation, as Datar et al. reported in cytotoxic T lymphocytes [47]. This view is consistent with the previous view that immune checkpoint molecules could serve as the markers of antigen-experienced T cells [56], as well as the view that prominent T-cell activation was followed by a dysfunctional phenotype characterized by engagement of apoptotic programs, which led to a vicious cycle of activation and apoptosis that limited anti-tumor immune efficacy [57]. Moreover, we made a combination and found that the low GZMB levels of NK cells independently predicted poor RFS, and the GZMB levels in TIM-3^+^ NK cells served as a superior indicator than in the total NK cells for relapse prediction. As we just discussed, TIM-3^+^ NK cells mark the full functional performance and maturity of NK cells. Correspondingly, TIM-3^+^ NK cells should exhibit a high expression of NK cell-associated cytotoxic molecules such as perforin and GZMB in order to fulfill the function. Thus, the TIM-3^+^ NK cells with low GZMB may have functional performance defects during maturation or may be over-matured NK cells that are approaching senescence or functional exhaustion. To our knowledge, this is the first time that cytotoxic molecule levels combined with TIM-3 expression in NK cells were applied to evaluating the prognostic significance.

There were several limitations in the current study. First, the clinical cohort study was retrospective, and therefore the patients’ treatment modalities were not fully uniform, which might have an influence on the survival analysis. Second, we did not perform scRNA-seq ourselves, and the scRNA-seq data we used were from the public database.

Taken together, through scRNA-seq analysis, fresh BM sample testing and in vitro stimulation assays, we displayed the positive correlation between TIM-3 expression and cytotoxic molecule levels in NK cells in both HDs and AML. It was cytotoxic molecule levels but not TIM-3 expression that positively correlated with NK-cell-killing activity against K562 cells. Both high TIM-3 expression and low GZMB levels on total NK cells of diagnostic BM specimens predicted poor clinical outcomes, and low GZMB levels in TIM-3^+^ NK cells independently predicted poorer RFS in AML. All in all, these results demonstrated that TIM-3 on NK cells did not just deliver co-inhibitory or co-stimulatory signals but were up-regulated expression upon encountering antigens. Thereafter, the binding of TIM-3 with its ligands further caused the engagement of pro-apoptosis programs, which eventually led to the dysfunction of NK cells as well as the weakening anti-tumor immunity and poor prognosis of AML. The results in the current study suggest the dynamic role of TIM-3 expression on NK cells, which is different from its function as a marker of the terminally dysfunctional subset and a co-inhibitory receptor on T cells in the microenvironment of multiple solid tumors [58,59,60]. Thus, the application of ICIs targeting TIM-3 should take the maturation stage of NK cells into account, for an optimal timing may exist in order to enhance the efficacy. Our study indicates a direction for the intervention timing of immune checkpoint inhibitors (ICIs) in the clinical application and triggers a novel thought on the failure of ICI therapy in AML.

## Figures and Tables

**Table 1:** The relationship between TIM-3 expression on NK cells and patients’ demographic and clinical characteristics in AML patients at diagnosis.

Variables	Number of Patients or Median (Range)	TIM-3 Expression on NK Cells (%)	*p* Value
All	105	68.9 (19.7–95.0)	
Age (y)	47 (16–65)		0.46
15–45	49, 46.7%	70.0 (21.8–95.0)	
45–65	56, 53.3%	68.1 (19.7–92.2)	
Gender			0.85
Male	55, 52.4%	69.1 (29.3–92.2)	
Female	50, 47.6%	67.7 (19.7–95.0)	
WBC count (×10^9^/L)	16.6 (1.2–460.0)		0.69
≤13	52, 50.0%	69.5 (19.7–92.2)	
>13	52, 50.0%	67.2 (32.6–95.0)	
Hemoglobin (g/L)	87 (36–152)		0.35
≤90	56, 53.8%	67.3 (19.7–95.0)	
>90	48, 46.2%	71.1 (29.3–92.1)	
Platelet count (×10^9^/L)	40 (4–507)		0.091
≤45	54, 51.9%	67.2 (19.7–92.1)	
>45	50, 48.1%	73.3 (30.7–95.0)	
BM blast (%)	61 (22–98)		0.51
≤60 (*n* = 50)	50, 47.6%	67.3 (21.8–92.1)	
>60 (*n* = 55)	55, 52.4%	73.4 (19.7–95.0)	
FAB subtypes			0.75
M0	3, 2.9%	77.6 (49.6–82.5)	
M1	5, 4.8%	78.7 (57.1–90.3)	
M2	64, 61.0%	69.1 (21.8–92.2)	
M4	27, 25.7%	65.5 (19.7–95.0)	
M5	5, 4.8%	73.1 (60.4–87.2)	
M7	1, 1.0%	55.9	
ELN genetic risk classification (*n* = 96) *			0.094
Favorable	44, 45.8%	66.4 (21.8–91.3)	
Intermediate	24, 25.0%	75.6 (19.7–95.0)	
Adverse	28, 29.2%	74.7 (44.3–92.2)	

* The ELN risk classification by genetics were defined based on the 2022 ELN guidelines.

**Table 2:** Univariate analysis and multivariate analysis of RFS (*n* = 70).

	Univariate Analysis		Multivariate Analysis		Multivariate Analysis *	
Variables	2-Year RFS (95% CI)	*p* Value	HR (95% CI)	*p* Value	HR (95% CI)	*p* Value
TIM-3/NK (%)		**0.013**		0.55		0.85
≤cutoff 72.0 (*n* = 40)	77.0 (48.9–90.9)		-		-	
>cutoff 72.0 (*n* = 30)	45.8 (21.4–67.4)		-		-	
GZMB/NK (%)		**0.0060**		**0.0020**		0.18
≤cutoff 77.2 (*n* = 39)	50.4 (28.4–68.8)		8.1 (2.2–30.7)		-	
>cutoff 77.2 (*n* = 31)	79.4 (51.9–92.2)		1.0		-	
GZMB/TIM3^+^NK (%) *		0.0026 *				**0.0032**
≤cutoff 84.0 (*n* = 36)	42.8 (18.9–64.9)				7.7 (2.0–30.0)	
>cutoff 84.0 (*n* = 34)	89.9 (71.9–96.7)				1.0	
GZMB/TIM-3^−^NK (%) *		0.0084 *				
≤cutoff 72.0 (*n* = 38)	53.9 (33.8–70.3)					0.65
>cutoff 72.0 (*n* = 32)	81.3 (44.7–94.8)				-	
Age (y)		0.99			-	
15–45 (*n* = 36)	68.6 (44.1–84.1)					
46–65 (*n* = 34)	61.9 (36.1–79.8)					
Gender		0.66				
Male (*n* = 37)	73.8 (54.1–86.0)					
Female (*n* = 33)	56.0 (27.8–76.8)					
WBC count (× 10^9^/L)		0.24				
≤13 (*n* = 36)	72.8 (51.2–86.0)					
>13 (*n* = 34)	44.7 (9.8–75.6)					
Hemoglobin (g/L)		0.51				
≤90 (*n* = 34)	59.2 (30.9–79.1)					
>90 (*n* = 36)	67.8 (41.7–84.2)					
Platelet count (× 10^9^/L)		0.33				
≤45 (*n* = 37)	64.4 (34.6–83.3)					
>45 (*n* = 33)	63.8 (41.6–79.4)					
BM blast (%)		0.43				
≤60 (*n* = 35)	66.4 (39.3–83.6)					
>60 (*n* = 35)	61.7 (35.2–80.0)					
ELN risk category by genetics (*n* = 67)		**0.11**		**0.0015**		**0.0018**
Favorable (*n* = 34)	71.7 (41.7–88.1)	-	1.0	-	1.0	-
Intermediate (*n* = 16)	55.1 (25.5–77.1)	0.030	11.3 (2.9–44.9)	**0.0006**	9.3 (2.6–32.4)	**0.0005**
Adverse (*n* = 17)	59.2 (23.7–82.6)	0.31	3.7 (1.1–12.9)	**0.040**	4.6 (1.3–16.6)	**0.021**
Induction therapy		0.54				
IA/HAA (*n* = 47)	56.1 (33.5–73.7)	-				
AA/CAG (*n* = 5)	75.0 (12.8–96.1)	0.67				
Azacitidine + Venetoclax (*n* = 16)	85.6 (53.3–96.2)	0.21				
Others (*n* = 2)	100	0.47				
CR after 1-course induction		0.73				
No (*n* = 11)	70.0 (22.5–91.8)					
Yes (*n* = 59)	63.9 (45.0–77.8)					
Consolidation therapy		**0.038**		**0.0020**		**0.0046**
Chemotherapy alone (*n* = 51)	56.8 (35.0–73.8)		12.9 (2.5–65.8)		10.4 (2.1–52.7)	
Allo-HSCT (*n* = 19)	84.0 (46.8–96.0)		1.0		1.0	

The bold values in univariate analysis mean variables with *p* value < 0.20 which were entered into multivariate analysis, and those in multivariable analysis mean significantly different. Variables with * were included in the multivariable analysis only once, and the corresponding results were displayed in the right side (marked with *) of the table.

**Table 3:** Univariate analysis and multivariate analysis of EFS (*n* = 77).

	Univariate Analysis		Multivariate Analysis		Multivariate Analysis *	
Variables	2-Year RFS (95% CI)	*p* Value	HR (95% CI)	*p* Value	HR (95% CI)	*p* Value
TIM-3/NK (%)		**0.0074**		0.31		0.31
≤cutoff 75.0 (*n* = 49)	63.4 (39.6–79.8)		-		-	
>cutoff 75.0 (*n* = 28)	39.3 (19.7–58.5)		-		-	
GZMB/TIM3^+^NK (%) *		0.0093 *				**0.072**
≤cutoff 87.95 (*n* = 48)	38.8 (19.7–57.5)				-	
>cutoff 87.95 (*n* = 29)	85.0 (64.7–94.1)				-	
Age (y)		0.92				
15–45 (*n* = 39)	60.9 (39.2–76.9)					
46–65 (*n* = 38)	50.8 (28.8–69.1)					
Gender		0.66				
Male (*n* = 40)	65.6 (47.2–79.0)					
Female (*n* = 37)	46.2 (23.1–66.4)					
WBC count (× 10^9^/L)		**0.19**		0.60		0.60
≤13 (*n* = 39)	62.4 (41.7–77.6)		-		-	
>13 (*n* = 38)	38.1 (9.4–67.4)		-		-	
Hemoglobin (g/L)		0.79				
≤90 (*n* = 38)	52.7 (28.1–72.4)					
>90 (*n* = 39)	54.6 (31.5–72.8)					
Platelet count (× 10^9^/L)		**0.035**		0.19		0.19
≤45 (*n* = 37)	62.3 (33.8–81.4)		-		-	
>45 (*n* = 40)	47.0 (27.8–64.1)		-		-	
BM blast (%)		**0.088**		0.67		0.67
≤60 (*n* = 36)	60.2 (35.0–78.2)		-		-	
>60 (*n* = 41)	50.3 (29.1–68.2)		-		-	
ELN risk category by genetics (*n* = 74)		**0.0044**		**0.0008**		**0.0008**
Favorable (*n* = 34)	71.7 (41.7–88.1)	-	1.0	-	1.0	-
Intermediate (*n* = 18)	43.0 (18.9–65.3)	**0.0011**	4.2 (1.4–12.5)	**0.0087**	4.2 (1.4–12.5)	**0.0087**
Adverse (*n* = 22)	38.8 (15.1–62.2)	**0.0061**	6.4 (2.2–18.4)	**0.0005**	6.4 (2.2–18.4)	**0.0005**
Induction therapy		**0.0004**		**0.0006**		**0.0006**
IA/HAA (*n* = 49)	50.4 (29.5–68.0)	-	1.0	-	1.0	-
AA/CAG (*n* = 5)	75.0 (12.8–96.1)	0.55	-	0.26	-	0.26
Azacitidine + Venetoclax (*n* = 16)	85.6 (53.3–96.2)	0.13	0.1 (0.01–0.8)	**0.027**	0.1 (0.01–0.8)	**0.027**
Other non-intensive chemotherapy regemens (*n* = 5)	20.0 (0.8–58.2)	**0.0006**	5.6 (1.7–18.2)	**0.0046**	5.6 (1.7–18.2)	**0.0046**

The bold values in univariate analysis mean variables with *p* value < 0.20 which were entered into multivariate analysis, and those in multivariable analysis mean significantly different.The variable with * was included in the multivariable analysis only once, and the corresponding results were displayed in the right side (marked with *) of the table.

## Data Availability

The data underlying this article are available from the corresponding author upon their reasonable request.

## Supplementary Materials

The following supporting information can be downloaded at: https://www.mdpi.com/article/10.3390/biomedicines12122717/s1, Table S1: The information of 16 AML patients used in single-cell RNAseq analysis; Figure S1: Quality control and primary clustering results of downstream data from single-cell RNA sequencing (scRNA-seq). The distribution of total RNA (nFeature_RNA), encoding RNA (nCount_RNA) and mitochondrial RNA proportion (percent.mt) of each bone marrow (BM) sample (A). The correlation between encoding RNA and mitochondrial RNA proportion as well as total RNA. Absolute values of the correlation coefficient lower than 0.1 between nCount_RNA and percent.mt, as well as higher than 0.8 between nCount_RNA and nFeature_RNA was considered scRNA-seq data to be well qualified (B). The primary clustering results based on the dimension reduction method “UMAP” with the integration function “harmony” and a dimension of 1:10 (C). Seurat “SignleR” annotation results showing that NK cells were enriched in the clusters 8 and 14 (D); Figure S2: The gating strategy of TIM-3 expression, Perforin (PFP) and Granzyme B (GZMB) levels of NK cells tested by multi-parameter flow cytometry (MFC) using fresh BM samples. Gating strategy of lymphocytes and TIM-3^+^, PFP^+^ and GZMB^+^ of lymphocytes (A). Gating strategy of NK cells and TIM-3^+^, PFP^+^ and GZMB^+^ of NK cells. The gates were linked with the internal controls of lymphocytes (B). Cross-shaped gating strategy of PFP and GZMB, TIM-3 and PFP, as well as TIM-3 and GZMB (C); Figure S3: The gating strategy of K562 apoptosis analysis using bone marrow mononuclear cells (BMMC) samples. K562 cells were circled out through different SS INT/SS PEAK from primary BMMCs (A). Annexin-V (Alexa Fluor 647) and 7-AAD were used to labelled apoptotic cells in Q2 and Q3 (B), Figure S4: The gating strategy (A-E) and cell purity examination (F-O) of TIM-3^+^ and TIM-3^−^ NK cells isolated by cell sorting based on flow cytometry. After doublets (A) and cell debris (B) were excluded, lymphocytes were circled out with CD45^high^/SSC^low^ (C), and then NK cells were defined as CD3^−^CD56^+^ in lymphocytes (D). TIM-3^+^ and TIM-3^−^ NK cells were individually collected circled with TIM-3high and TIM-3low gated on NK cells (E). All collected TIM-3^+^ (F) and TIM-3^−^ (K) NK cells were tested purity. Dead cells and cell debris were removed using CD45 staining (G, L). CD3/CD56 circling using All cells were applied to showing the gating boundary of CD3^−^CD56^+^ as an internal control (H, M), and NK cells gated on lymphocytes were then circled out linked to this gating boundary (I, N). TIM-3^+^ and TIM-3^−^ cells were circled out gated on NK cells (J, O). The purity of TIM-3^+^ was 80.5%, and that of TIM-3^−^ was 77.7% in this case; Figure S5: The Kaplan-Meier survival curves of TIM-3 expression, PFP and GZMB levels of NK cells in AML based on a preliminary analysis using the quartile of each indicator. RFS and EFS curves of TIM-3 (A, D), PFP (B, E) and GZMB (C, F) levels of NK cells. RFS and EFS curves of PFP and GZMB levels individually in TIM-3^+^ (G, K, I, M) and TIM-3^−^ (H, L, J, N) NK cells; Figure S6: The ROC analysis for variables showing correlated tendency with RFS and EFS. ROC analysis of TIM-3 expression and GZMB levels of NK cells based on relapse or not (A, B), and of TIM-3 expression on NK cells based on event occurrence or not (C). ROC analysis of PFP (D) and GZMB (F) levels in TIM-3^−^ NK cells, as well as GZMB in TIM-3^+^ NK cells (E) based on relapse or not, and of PFP in TIM-3^−^ NK cells (G) and GZMB in TIM-3^+^ NK cells (H) based on event occurrence or not.
