# Supplementary material for: The Functional Role and Prognostic Significance of TIM-3 Expression on NK Cells in the Diagnostic Bone Marrows in Acute Myeloid Leukemia
Source: Biomedicines. 2024 Nov 27;12(12):2717. doi: 10.3390/biomedicines12122717 (PMC11727352; doi:10.3390/biomedicines12122717)
Supplement: Supplementary file 1 [file biomedicines-12-02717-s001.zip › biomedicines-3312763-supplementary.pdf]

Table S1. The information of 16 AML patients used in single-cell RNAseq analysis.

Sample	Days from diagnosis	Gender	Age	RHP Mutations	Cytogenetics	Common translocation	Remarks
AML1012	D0	F	32	KRAS NM_004985 c.38G>A p.G13D (4.6%) /// NRAS NM_002524 c.38G>A p.G13D (39.0%) /// NOTCH2 NM_024408 c.4238T>A p.L1413H (50.8%, VUS) /// SF3A1 NM_005877 c.1432G>A p.G478S (44.2%, VUS)	46,XX,inv(16)(p13q22)[4]/48,idem,+8,+21[16]	CBFB-MYH11	Newly diagnosed AML, pre-treatment.
AML210A	D0	M	67	DNMT3A NM_175629 c.2644C>T p.R882C (43.4%) /// NPM1 NM_002520 c.859_860insTCTG p.W288fs*>9 (42.7%) /// TET2 NM_001127208 c.1072A>G p.S358G (49.3%, VUS) /// FLT3-ITD NM_004119 c.1802_1802insTTGAATATGATCTCAAATGGGAGTTTCCAAGAGAAAATTTAGAGTTTG	46,XY[20]	NA	Newly diagnosed AML with myelomonocytic differentiation, pre-treatment.
AML419A	D0	F	54	CEBPA NM_004364 c.118_118insC p.P39fs* (42.9%) /// DNMT3A NM_175629 c.2644C>T p.R882C (41.9%) /// NPM1 NM_002520 c.859_860insTCTG p.W288fs*>9 (37.9%) /// FLT3 NM_004119 c.2039C>T p.A680V (29.0%) /// FLT3 NM_004119 c.2523C>A p.N841K (16.2%) /// FLT3-ITD NM_004119 c.1819_1819insTATGATCTCAAATGGGAGTTTCCA (13.5%) /// JAK3 NM_000215 c.2773C>A p.R925S (55.1%, VUS)	46,XX[20]	NA	Newly diagnosed AML with monocytic differentiation, pre-treatment.
AML916	D0	F	57	TP53 NM_000546 c.713G>A p.C238Y (97.6%)	46,XX[20]	NA	Mixed phenotype acute leukemia expressing markers of stem cells (CD34, CD117), myeloid (CD64), T (CD3) and B (CD19) lineages by flow, pre-treatment.
AML921A	D0	M	42	DNMT3A NM_175629 c.2645G>A p.R882H (44.2%) /// RUNX1 NM_001754 c.167T>C p.L56S (63.5%, VUS) /// SETD2 NM_014159 c.3229A>G p.T1077A (48.4%, VUS)	46,XY	NA	Newly diagnosed AML, pre-treatment.
AML314	D0	M	54	BCOR NM_001123385 c.2098delG p.K699fs* (36.4%) /// RUNX1 NM_001754 c.966_967delTC p.S322fs*160 (17.2%)	46,XY	NA	Newly diagnosed AML NOS, pre-treatment.
AML314	D31	M	54	Not performed	46,XY[20]	NA	Morphologic remision, post-induction chemotherapy.
AML371	D0	M	51	NRAS NM_002524 c.181C>A p.Q61K (37.9%) /// WT1 NM_024426 c.1130_1130insGTAGCCCCGA p.T309fs*11 (13.3%)	46,XY,der(16)t(16;18)(p1?2;p11.3)del(16)(q22q24), der(18)t(16;18)(p12;p11.3)[20]. ish der(16)(18pter+,5'CBFB+), der(18)(pter-)	NA	Newly diagnosed AML with monocytic differentiation, pre-treatment.
AML371	D34	M	51	None Detected	46,XY	NA	Morphologic remision, post-induction chemotherapy.
AML475	D0	M	70	DNMT3A NM_175629 c.2645G>A p.R882H (43.9%) /// BCOR NM_001123385 c.2926C>T p.R976* (83.0%) /// BCORL1 NM_021946 c.1942_1943insC p.T648fs* (13.3%) /// BCORL1 NM_021946 c.2996delC p.T999fs* (8.2%) /// BCORL1 NM_021946 c.3142C>T p.R1048* (50.0%) /// BCORL1 NM_021946 c.3586C>T p.R1196* (4.1%)	46,XY	NA	Newly diagnosed AML with monocytic differentiation, pre-treatment.
AML475	D29	M	70	Not performed	46,XY[20]	NA	Morphologic remission, post-induction chemotherapy.
AML722B	D0	F	52	BCORL1 NM_021946 c.1627delG p.D542fs* (7.2%) /// IDH2 NM_002168 c.515G>A p.R172K (42.3%) /// ASXL1 NM_015338 c.1231C>T p.R411C (45.3%, VUS) /// PHF6 NM_001015877 c.976T>C p.Y325H (38.2%, VUS) /// PTPN11 NM_002834 c.893A>G p.N298S (51.2%, VUS)	46,XX,i(7)(p10) or add(7)(q11.2)[17]/47,XX,+8[3]	NA	Newly diagnosed AML, pre-treatment.
AML722B	D49	F	52	IDH2 NM_002168 c.515G>A p.R172K (0.5%) /// ASXL1 NM_015338 c.1231C>T p.R411C (46.7%, VUS) /// PTPN11 NM_002834 c.893A>G p.N298S (47.4%, VUS)	Unknown	NA	Post 7+3 induction and 2+5 re-induction chemotherapy.
AML870	D0	M	32	ZRSR2 NM_005089 c.1147C>G p.P383A (99.6%, VUS)	46,XY,t(9;11)(p21;q23)[8].nuc ish(MLLx2)(5' MLL sep 3'MLLx1)[91/100]	MLL-X	Newly diagnosed AML with recurrent genetic abnormalities, pre-treatment.
AML870	D14	M	32	Not performed	Not performed	NA	Ablated, post-induction chemotherapy.
AML997	D0	M	62	DNMT3A NM_175629 c.2645G>A p.R882H (43%) /// NPM1 NM_002520 c.859_860insTCTG p.W288fs*>9 (46%) /// CEBPA NM_004364 c.138insT p.A47fs (48%) /// FLT3-ITD (exon 14 ITD)	46,XY	NA	Newly diagnosed AML with monocytic differentiation.
AML997	D35	M	62	Not performed	46,XY[20]	NA	Morphologic remission, post-induction chemotherapy.
AML329	D0	F	73	NPM1 NM_002520 c.859_860insTCTG p.W288fs*>9 (49.3%) /// NOTCH1 NM_017617 c.5273G>A p.R1758H (67.9%, VUS) /// SMC3 NM_005445 c.3449A>G p.D1150G (45.1%, VUS) /// FLT3-ITD NM_004119 c.1800_1800insCTACGTTGATTTCAGAGAATATGA	46,XX[20]	NA	Newly diagnosed AML with monocytic differentiation, pre-treatment.
AML329	D20	F	73	Unknown	Unknown	NA	Post 7+3 induction chemotherapy, ablated marrow.
AML329	D37	F	73	Unknown	Unknown	NA	Remission.
AML420B	D0	M	58	IDH2 NM_002168 c.419G>A p.R140Q (23.2%) /// TP53 NM_000546 c.818G>T p.R273L (16.0%) /// SH2B3 NM_005475 c.1655A>G p.D552G (55.7%, VUS)	46,XY,add(1)(p36.1)[3]/46,XY[17]	NA	Newly diagnosed AML, pre-treatment. Possible plasma cell neoplasm in the background.

AML420B	D14	M	58	Unknown	Unknown	NA	Post 7+3 induction chemotherapy.
AML420B	D35	M	58	Unknown	45,X,-Y,add(1)(p36.1)[1]/46,XY[19]	NA	
AML556	D0	M	70	DNMT3A NM_175629 c.2644C>T p.R882C (43.5%) /// NPM1 NM_002520 c.859_860insTCTG p.W288fs*>9 (35.8%) /// NRAS NM_002524 c.183A>T p.Q61H (43.8%) /// NRAS NM_002524 c.35G>A p.G12D (3.8%) /// TET2 NM_001127208 c.3176C>G p.S1059* (37.0%) /// TET2 NM_001127208 c.5412_5413insA p.L1804fs* (34.4%) /// ATM NM_000051 c.6067G>A p.G2023R (51.9%, VUS)	46,XY	NA	Newly diagnosed AML with myelomonocytic differentiation, pre-treatment. Also diagnosed with smoldering myeloma at the same time.
AML556	D15	M	70	Not performed	Not performed	NA	Ablated, post-induction chemotherapy.
AML556	D31	M	70	Not performed	46,XY[20]	NA	Morphologic remission, post-induction chemotherapy.
AML328	D0	F	74	DNMT3A NM_175629 c.1910T>A p.L637Q (43.9%) /// TP53 NM_000546 c.431A>C p.Q144P (38.7%, VUS) /// TP53 NM_000546 c.455C>G p.P152R (51.5%) /// FLT3-ITD NM_004119 c.1749_1752delCTCCinsAGGTCAG p.584_585delSinGQ	45,XX,ider(3)(q10)inv(3)(q21q26.2),add(5)(q13),-7,add(9)?dup(q13q22)[19]/ 46,XX[1].ishider(3)(RP11-669C7/RP11-637O11 sep,RP11-82C9,RP11-362K14+)x2[5]	NA	Newly diagnosed AML, pre-treatment.
AML328	D29	F	74	DNMT3A NM_175629 c.1910T>A p.L637Q (28.1%) /// TP53 NM_000546 c.431A>C p.Q144P (44.0%, VUS) /// TP53 NM_000546 c.455C>G p.P152R (20.6%) /// FLT3-ITD NM_004119 c.1749_1752delCTCCinsAGGTCAG p.584_585delSinGQ (9.5%)	45,XX,ider(3)(q10)inv(3)(q21q26.2),add(5)(q13),-7,add(9)?dup(q13q22)[7]/45,idem,add(16)(q13)[cp3]	NA	Azacitidine + venetoclax, C1D27.
AML328	D113	F	74	DNMT3A NM_175629 c.1910T>A p.L637Q (26.7%) /// TP53 NM_000546 c.431A>C p.Q144P (34.6%, VUS) /// TP53 NM_000546 c.455C>G p.P152R (11.3%) /// FLT3-ITD NM_004119 c.1749_1752delCTCCinsAGGTCAG p.584_585delSinGQ (3.4%)	45,XX,ider(3)(q10)inv(3)(q21q26.2),add(5)(q13),-7,dup(9)(q13q22)[1]/46,XX[1].nuc ish(D5S723/D5S721x2,EGR1x1)[5/100]	NA	Azacitidine + venetoclax, C4D23.
AML328	D171	F	74	Unknown	45~46,XX,ider(3)(q10)inv(3)(q21q26.2),add(5)(q13),-7,add(9)?dup(9)(q13q22),add(17)(p11.2)[4], +mar[5][cp13]/43~44,idem,add(3)(q12)[2],der(9)t(9;9)(p22;q13),der(15;16)(q10,q10),-17,del(18)(q?21)[2],+mar[2][cp7]	NA	Azacitidine + venetoclax, C6D17.
AML707B	D0	M	26	BRCC3 NM_024332 c.686_687insTGATGTCGCG p.L229fs* (77.0%) /// KIT NM_000222 c.2468A>G p.Y823C (32.8%) /// RAD21 NM006265 c.1058_1058insCC p.L353fs* (39.7%)	45,X,-Y,t(8;21)(q22;q22)[10]/45,idem,t(2;5)(p21;q31)[9]/46,XY[1]	RUNX1-RUNX1T1	Newly diagnosed AML, pre-treatment.
AML707B	D18	M	26	Unknown	Unknown	NA	Day 17 post 7+3 induction chemotherapy.
AML707B	D41	M	26	None Detected	46,XY[20]	NA	Day 40 post 7+3 induction chemotherapy.
AML707B	D97	M	26	Unknown	46,XY[20]	NA	Day 38 post high-dose Ara-C consolidation (HIDAC), C1D38.
AML707B	D113	M	26	Unknown	Unknown	NA	Day 54 post HIDAC, C1D54.

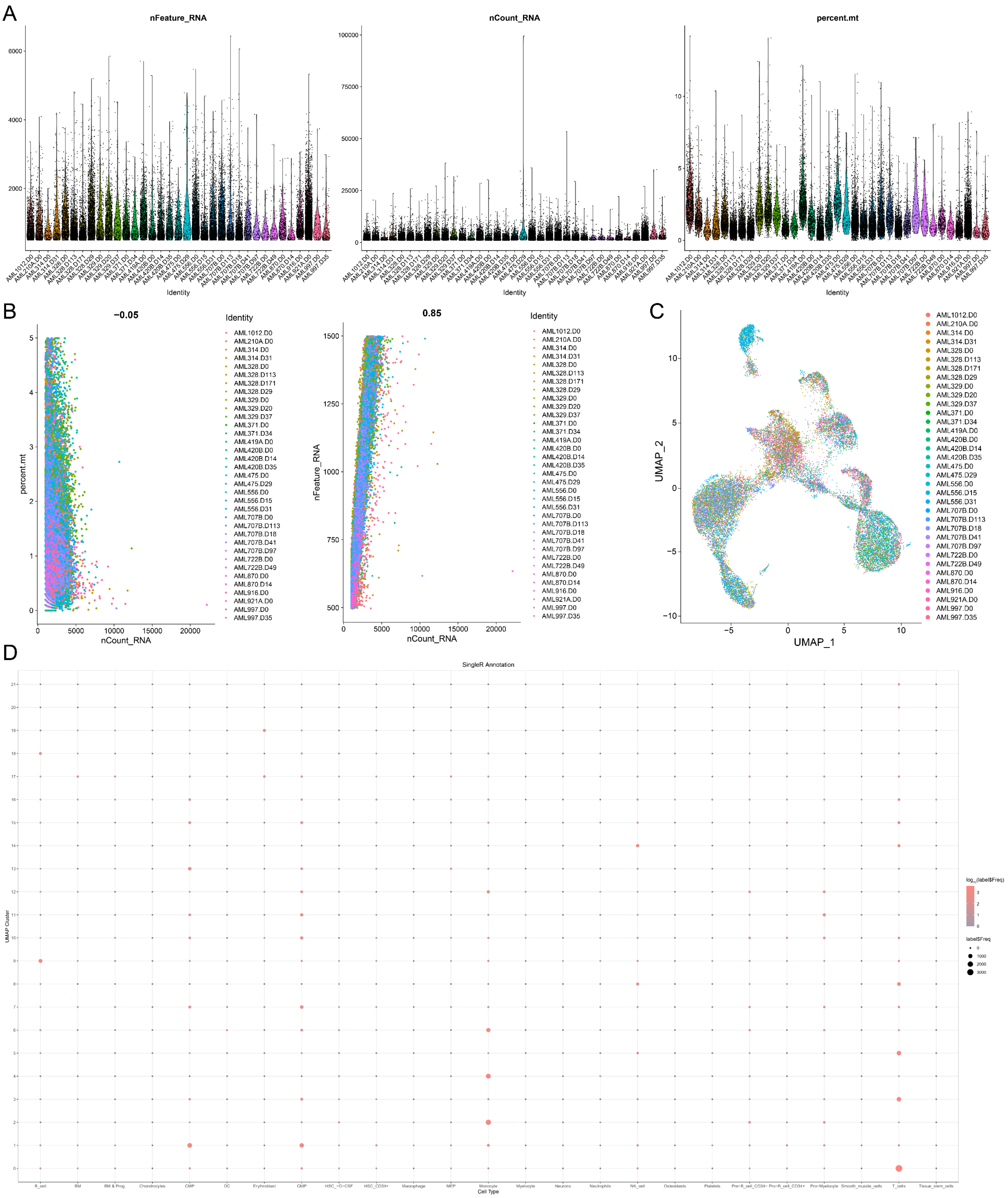

Figure S1. Quality control and primary clustering results of downstream data from single-cell RNA sequencing (scRNA-seq). The distribution of total RNA (nFeature_RNA), encoding RNA (nCount_RNA) and mitochondrial RNA proportion (percent.mt) of each bone marrow (BM) sample (A). The correlation between encoding RNA and mitochondrial RNA proportion as well as total RNA. Absolute values of the correlation coefficient lower than 0.1 between nCount_RNA and percent.mt, as well as higher than 0.8 between nCount_RNA and nFeature_RNA was considered scRNA-seq data to be well qualified (B). The primary clustering results based on the dimension reduction method “UMAP” with the integration function “harmony” and a dimension of 1:10 (C). Seurat “SignleR” annotation results showing that NK cells were enriched in the clusters 8 and 14 (D).

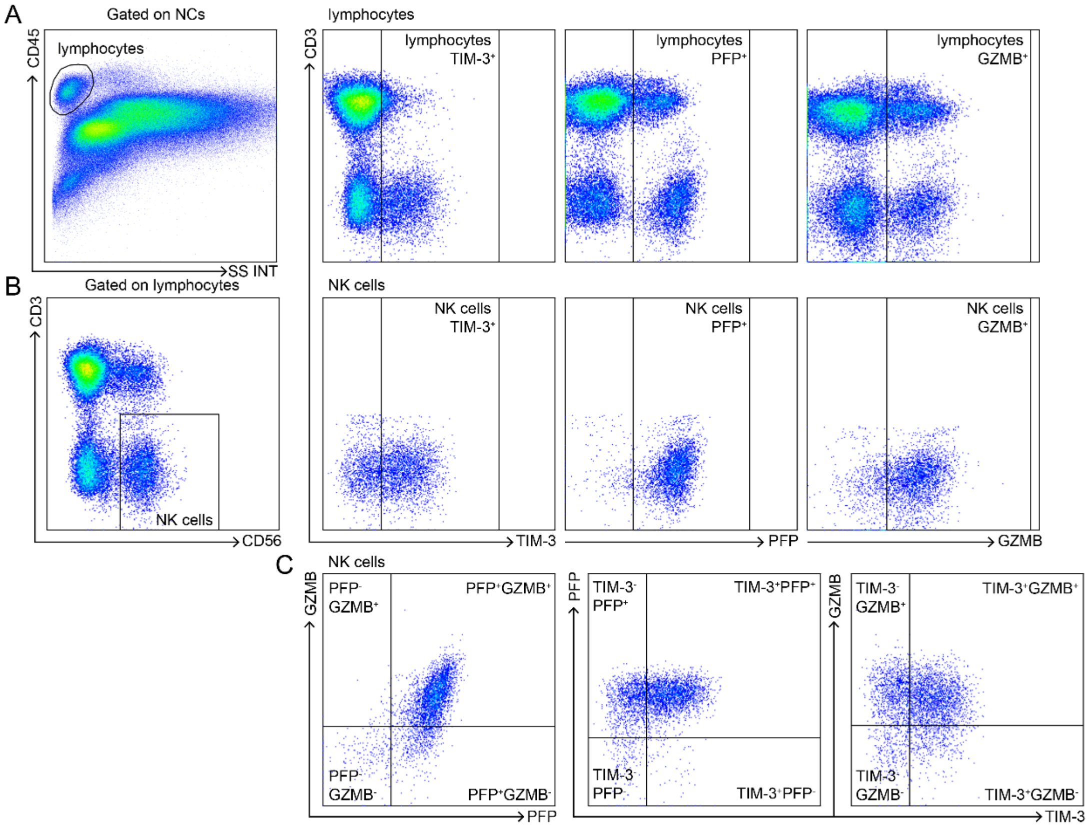

Figure S2. The gating strategy of TIM-3 expression, Perforin (PFP) and Granzyme B (GZMB) levels of NK cells tested by multi-parameter flow cytometry (MFC) using fresh BM samples. Gating strategy of lymphocytes and TIM-3⁺, PFP⁺ and GZMB⁺ of lymphocytes (**A**). Gating strategy of NK cells and TIM-3⁺, PFP⁺ and GZMB⁺ of NK cells. The gates were linked with the internal controls of lymphocytes (**B**). Cross-shaped gating strategy of PFP and GZMB, TIM-3 and PFP, as well as TIM-3 and GZMB (**C**).

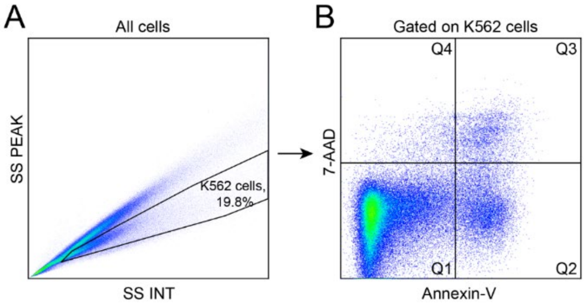

Figure S3. The gating strategy of K562 apoptosis analysis using bone marrow mononuclear cells (BMMC) samples. K562 cells were circled out through different SS INT/SS PEAK from primary BMMCs (**A**). Annexin-V (Alexa Fluor 647) and 7-AAD were used to labelled apoptotic cells in Q2 and Q3 (**B**).

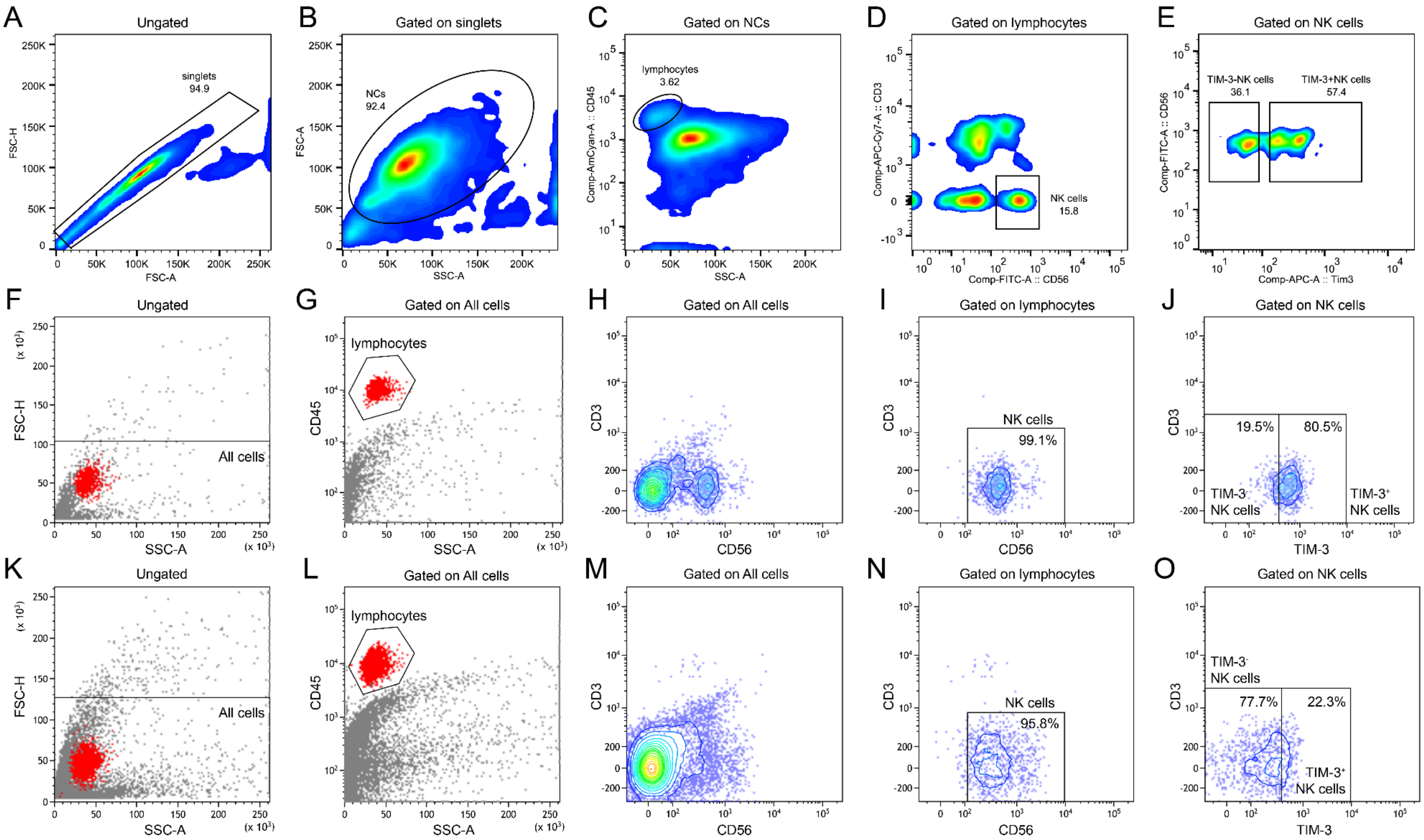

Figure S4. The gating strategy (**A-E**) and cell purity examination (**F-O**) of TIM-3$^+$ and TIM-3$^-$ NK cells isolated by cell sorting based on flow cytometry. After doublets (**A**) and cell debris (**B**) were excluded, lymphocytes were circled out with CD45^{high}/SSC^{low} (**C**), and then NK cells were defined as CD3$^-$CD56$^+$ in lymphocytes (**D**). TIM-3$^+$ and TIM-3$^-$ NK cells were individually collected circled with TIM-3^{high} and TIM-3^{low} gated on NK cells (**E**). All collected TIM-3$^+$ (**F**) and TIM-3$^-$ (**K**) NK cells were tested purity. Dead cells and cell debris were removed using CD45 staining (**G, L**). CD3/CD56 circling using All cells were applied to showing the gating boundary of CD3$^-$CD56$^+$ as an internal control (**H, M**), and NK cells gated on lymphocytes were then circled out linked to this gating boundary (**I, N**). TIM-3$^+$ and TIM-3$^-$ cells were circled out gated on NK cells (**J, O**). The purity of TIM-3$^+$ was 80.5%, and that of TIM-3$^-$ was 77.7% in this case.

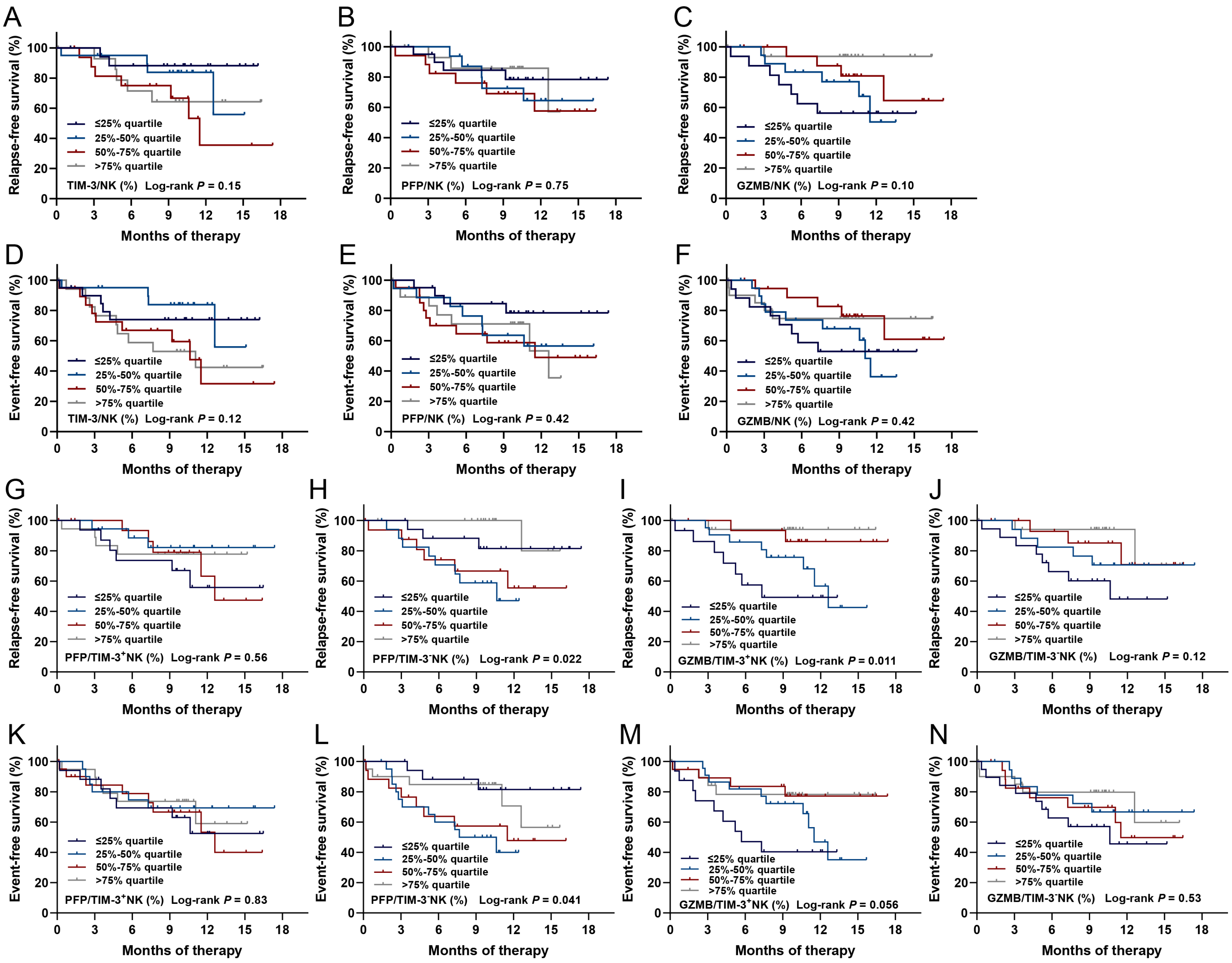

Figure S5. The Kaplan-Meier survival curves of TIM-3 expression, PFP and GZMB levels of NK cells in AML based on a preliminary analysis using the quartile of each indicator. RFS and EFS curves of TIM-3 (**A, D**), PFP (**B, E**) and GZMB (**C, F**) levels of NK cells. RFS and EFS curves of PFP and GZMB levels individually in TIM-3$^+$ (**G, K, I, M**) and TIM-3$^-$ (**H, L, J, N**) NK cells.

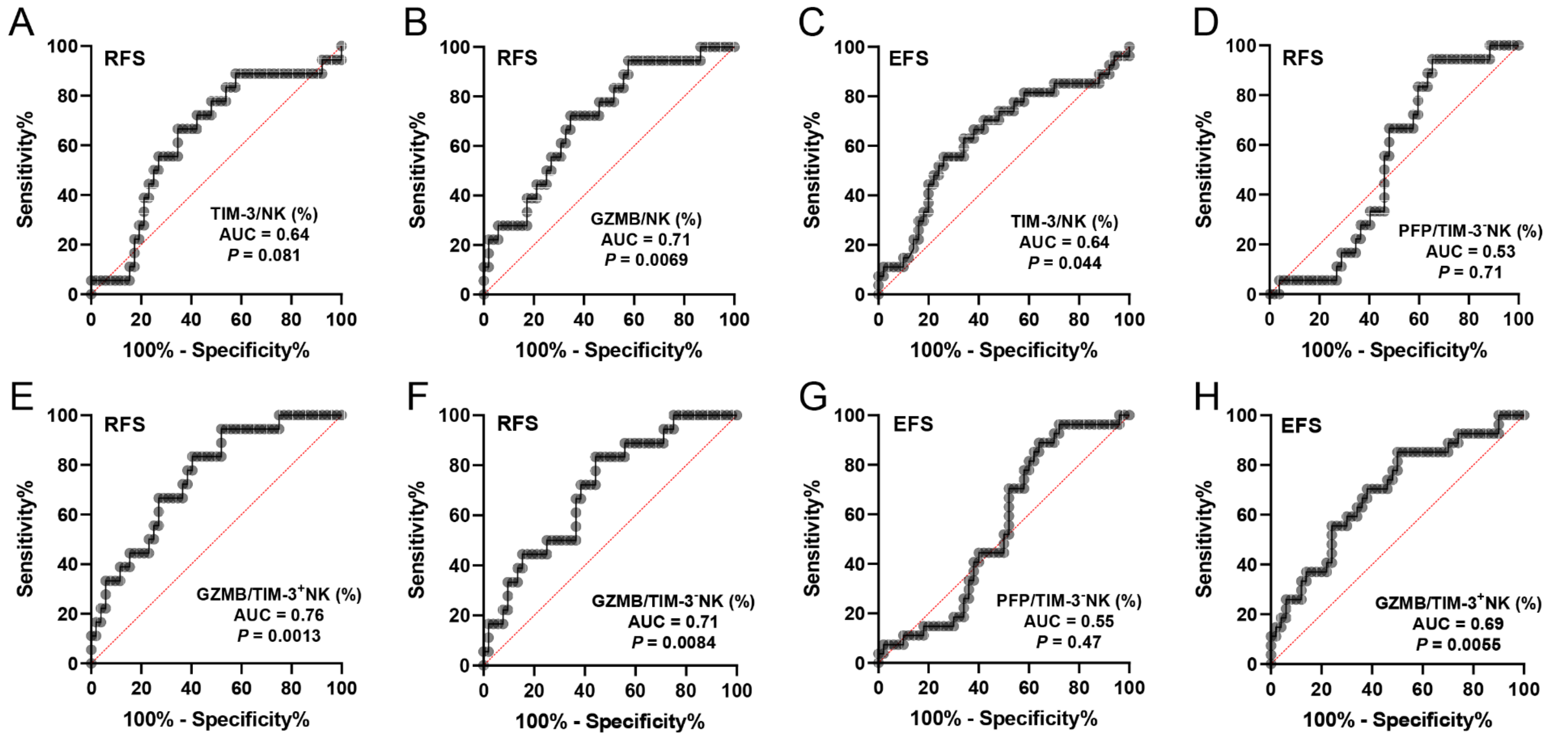

Figure S6. The ROC analysis for variables showing correlated tendency with RFS and EFS. ROC analysis of TIM-3 expression and GZMB levels of NK cells based on relapse or not (**A, B**), and of TIM-3 expression on NK cells based on event occurrence or not (**C**). ROC analysis of PFP (**D**) and GZMB (**F**) levels in TIM-3$^-$ NK cells, as well as GZMB in TIM-3$^+$ NK cells (**E**) based on relapse or not, and of PFP in TIM-3$^-$ NK cells (**G**) and GZMB in TIM-3$^+$ NK cells (**H**) based on event occurrence or not.
